# Evidence of the exploitation of marine resource by the terrestrial insect *Scapteriscus didactylus *through stable isotope analyzes of its cuticle

**DOI:** 10.1186/1472-6785-6-6

**Published:** 2006-05-08

**Authors:** Alexandra Maros, Alain Louveaux, Caroline Lelarge, Marc Girondot

**Affiliations:** 1 Laboratoire d'Ecologie, Systématique et Evolution (UMR 8079) Bât. 362 Université Paris-Sud, Orsay 91405 Cedex, France; 2 Institut de Biotechnologie des Plantes, Laboratoire Structure et Métabolisme des Plantes (UMR-CNRS 8618) Bât. 630 Université Paris-Sud, Orsay 91405 Cedex, France

## Abstract

**Background:**

About 4 × 10^5 ^eggs in more than 5000 marine turtle nests are deposited every year on a 3.6 km long beach in French Guiana (South America). The dry biomass of eggs is estimated to be 5 × 10^3 ^kg, yet only 25% of this organic matter will return to the ocean in the form of hatchlings. Such amounts of organic matter are supposed to drive the functioning of the beach ecosystem. Previous studies have shown that egg predators and detritivorous organisms dominate the trophic relationships and the dynamics of the system. The role of a terrestrial insect *Scapteriscus didactylus *(Latreille), which damages up to 40% of the eggs of the marine turtle (*Dermochelys coriacea*), was unexpected. However it was impossible from direct observations to prove that the mole cricket consumed a significant amount of these eggs. Therefore, the precise place of the mole cricket in the nitrogen and carbon cycles of the beach ecosystem could not be determined. In order to answer this question, we looked for a marine signature of carbon and nitrogen source metabolized by the mole cricket.

**Results:**

This study estimated the individual variability of δ^13^C and δ^15^N in the cuticle of *Scapteriscus didactylus*. The isotopic signature was compared between individuals collected at two sites: a village where mole crickets fed on human food scraps and the nearby Awala-Yalimapo beach, where food availability depends seasonally on the nesting sea turtles. The mole crickets collected near the habitations garbage showed no significant variations in the stable isotopic signature, within-and between age groups. On the contrary, isotopic values shifted from a signature of a terrestrial herbivorous diet in the mole crickets during early developmental stages, to isotopic values in adults in accordance with the exploitation of marine animal resources.

**Conclusion:**

The heterogeneity of individual signatures during the year is due to a selective exploitation of the food sources, differing in space and time. Some individuals, from the beach sample consumed a sufficient quantity of turtle eggs to induce the increase of isotopic enrichment observed in the cuticle. *Scapteriscus didactylus *is an opportunist feeder and plays a role in the turn over of the beach organic matter.

## Background

In French Guiana (South America), about 4 × 10^5 ^eggs in more than 5000 turtle nests are laid every year over a 3.6 km stretch of beach in the Awala-Yalimapo villages. The nesting season of the leatherback turtle (*Dermochelys coriacea*) takes place from March to July, peaking in May. The females come ashore at night and dig a nest 60–80 cm deep in the sand, at the boundary of the high tide mark and vegetation limit. Each nest contains about 80–90 eggs that hatch two months later. This large amount of nutrients and energy is exploited by vertebrates (dog, black vulture or man) and invertebrates (mole crickets *Scapteriscus didactylus*). Mole crickets, have been observed to pierce a hole in the turtle eggs, causing damage to an average of 18% (range 3.6 to 40%) of yolked eggs in the nests [1]. On a nesting beach in Florida, Bouchard and Bjorndal [2] estimated that only 25% of the organic matter introduced into nests by loggerhead turtles returned to the ocean as hatchling, and 29–40% of all nutrients were available to decomposers. Such studies only quantitatively assess egg predation by carnivores is (e.g. [3]). Assessing the importance of the omnivores is not as easy, particularly when direct observations and analyzes of fecal pellets give limited information (e.g. [4,5]). Assigning a diet at the species or population level leads to simplifications and ignores individual variations that exist in every population [6]. In such a view, a population could be composed of individuals of different ages, each of which may prefer and perform better on a particular subset of the resources [7].

The mole cricket *Scapteriscus didactylus *is an under ground insect, active at night. As such, its feeding behavior is difficult to investigate, a task not made any easier by the fact that very few identifiable remains are found in the digestive tract. *S. didactylus *is an introduced species, native of South America, to the Caribbean islands and the south coast of Australia. It lives inland and causes damages to crops (e.g. [8-10]). The life cycle of *S. didactylus *is likely to have two overlapping generations per year. Juveniles hatch in March and April become adults in June and July. This first generation develops during the nesting season of the turtles. The second generation develops from September to October, becoming adults in January and February [11]. Overlapping generations have been also observed in the Amazon basin for *S. didactylus *and *Neocurtilla hexadactyla *but more observations are needed [12]. The biology of *S. didactylus *has been studied in relation to the damages inflicted on golf lawns and the species was initially described as being mainly herbivorous, feeding on leaves and roots ([13,14]). However, analysis of the gut content of individuals collected on beaches of the Dominican Republic, indicate that this species may be carnivorous [9]. As such, the diet of *S. didactylus *appears to be omnivorous at the species level. A previous study [1] could not determine if the mole crickets ate a significant amount of the turtle eggs. In order to quantify the mole cricket's contribution to the nitrogen and carbon cycles of the beach, we analyzed the cuticle of adults and juveniles for enrichment in the stable carbon and nitrogen isotopes.

Stable isotopic signatures in animal tissues can be used to study food webs (e.g. [15,16]), especially to distinguish between terrestrial and marine nitrogen sources (see [5,16,17]). Leatherback turtles feed upon jellyfish, salps and other gelatinous organisms [18]. Studying the isotopic signatures of three marine turtles, Godley *et al*. [17] showed that the leatherback turtle occupies an intermediate level in the marine trophic web between the herbivorous green turtle (*Chelonia mydas*) and the carnivorous loggerhead turtle (*Caretta caretta*). Hence isotopic enrichment of the turtle egg proteins exhibited high δ^15^N; values 6‰ and 12‰ were observed for the eggs of green and loggerhead turtles respectively [19]. We looked for such a signature in the cuticle of the mole crickets, and compared these to the signature values measured in the cuticle of mole crickets living inland. Populations of *S. didactylus *were discovered in the cooking area of the village habitations. Fishermen houses are a few hundred meters back from the beach and mole crickets concentrate in areas where inhabitants cook and leave food scraps.

The cuticle of insects has a fast turnover with tissue being replaced at each moult. Mole crickets pass through 8 or 9 instars before the imaginal moult. At each moult, the old cuticle is digested and some of the materials present in the cuticle are conserved. Chitin is rebuilt in long chains of a polysaccharide made up largely of N-acetyleglucosamine. It is associated with protein to form a glycoprotein metabolized during the preceding instar. Fifteen to twenty days after the imaginal moult, the cuticle does not grow anymore [20]. Isotopic composition of the cuticle of an adult has the potential to provide an understanding of what the insect consumed during its juvenile stages irrespective of its diet in adulthood.

## Results

### (1) Stable isotopic content (δ^15^N and δ^13^C) of the potential food resources

Isotope signatures of the potential foods eaten by the mole crickets are presented in table 1. On the beach, the dicotyledons *Ipomoeae *spp. are the dominant plants which stay green all the year. At the top of the beach, grasses and sedges are wilted from August and the preceding five months of the dry season. The range of the δ^15^N values in herbaceous roots (3‰ and 6.5‰) is much lower than the mean value of leatherback eggs (10‰). Dicotyledon roots were found to be less enriched in ^13^C (-26‰ – -27‰) than grass roots (-16‰). Albumin of turtle eggs showed a higher enrichment in ^13^C (-17‰) than the yolk (-23‰).

Mole crickets living in the habitation garbage feed on rice and cassava, both of which have low nitrogen content; as a consequence δ^15^N values for cassava were undetectable (Table 1). At the habitations, enrichment in ^15^N of the mole cricket is mainly due to chicken meat (2‰) and fish scraps (13‰).

### (2) Mean isotopic enrichment between sampling locations

**Figure 1:**
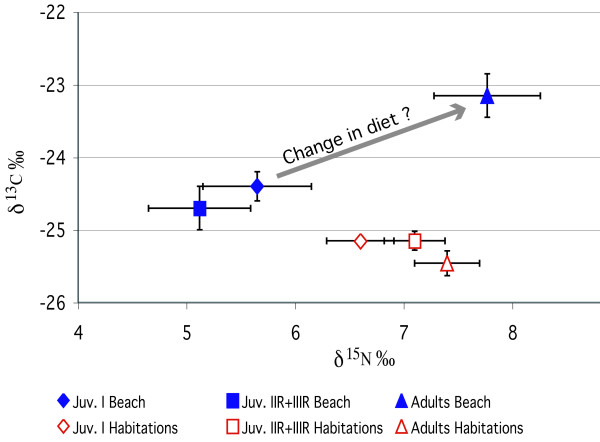
Carbon and nitrogen stable isotopes (δ^13^C and δ^15^N). Mean ratios measured on the cuticle of juvenile and adult mole cricket forelegs collected on the Awala-Yalimapo beach and from garbage at habitations. Mean ± Standard Error.

The mean stable isotopic ratio measurements in the cuticle are interpreted as isotopic signatures of the dietary history of the insect during the previous stages of development, and are compared between the two sample locations. At the habitation sites the mole crickets live in the cooking areas. Their diet is dependent on the foodstuffs cooked during the year. We observed a narrow range of variation of the isotopic values (Table 2; Figure 1) and analysis of the variance on δ^15^N and δ^13^C showed no significant difference in the ratio between juveniles and adults (respectively *F *_2,63 _= 0.94; *P *= 0.40 and *F *_2,63 _= 0.91; *P *= 0.41).

In the beach sample, carbon and nitrogen isotopic ratio have a wider range. Standard deviations in the samples increased between juveniles and adults (Table 2). A significant difference for δ^15^N and δ^13^C was found between the different developmental stages (*F *_2,97 _= 4.47; *P *= 0.01 and *F *_2,97 _= 4.83; *P *= 0.01 respectively).

A Welch's t-test (corrected for unequal variances) conducted on the data from animals sampled on the beach (Table 2) showed that the δ^13^C mean ratio was significantly higher for adults than for juveniles IIR + IIIR (-23.14 and -24.74 respectively) *t *= 3.88; *p *< 0.001; *dl *= 56.9. Furthermore, comparing adults from the beach and from habitations, the δ^13^C mean ratio was also significantly higher (-23.14 and -25.46 respectively) *t *= 6.91; *p *< 0.001; *dl *= 101.4. These results, combined with the high variability of the data, could reflect a change in the diet of the older larvae, which settled in the beach (juv. IIIR) before they became adults (Figure 1).

### (3) Temporal variations of the isotopic ratios

**Figure 2:**
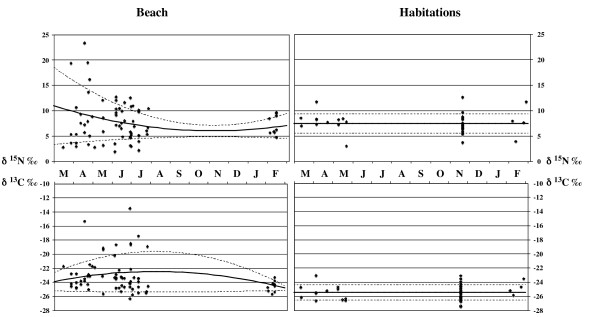
Selected models for δ^13^C and δ^15^N from cuticle of mole crickets caught on the beach or near habitations. The envelope is the selected model for ± 2 SD.

To understand the isotopic signature shift at the end of the development, adult data were re-analyzed taking into account the date of capture. Model selection is shown on Annex. Models with constant δ^15^N and δ^13^C values according to Julian date are selected for individuals caught at the habitation site, whereas second order polynomial models are selected for individuals caught on the beach (Figure 2). The selected model for standard deviation (SD) is very different between individuals caught at the habitations (constant SD) and individuals caught on beach (SD variable). A single model for both individuals caught on the beach and near habitation is strongly rejected compared to a specific model for each location (see annex, column Common model).

When the turtle eggs are present on the beach (March to July), the δ^13^C and δ^15^N signatures of the crickets are much more variable than outside the nesting season (August to February). After which it reaches the same value as for mole crickets sampled from the habitations, for which no temporal variation is detected (Figure 2).

The total contribution of leatherback turtle nests to the beach ecosystem was measured as kg of organic matter per month. This biomass is significantly correlated with the δ^13^C signature of mole crickets caught in the beach (Spearman non-parametric rank correlation, *p corrected for ex-aequo *= 0.02) but not with the δ^13^C signature of mole crickets from habitations (Spearman non-parametric rank correlation, *p corrected for ex-aequo *= 0.84). δ^15^N signatures for both habitation and beach sites are not significantly correlated with the mass of organic matter from marine origin deposited on the beach by marine turtles (*p *= 0.88 and *p *= 0.09 respectively).

## Discussion

Stable isotope studies, involving insects are generally investigated using the whole body or muscles [4]. Muscle tissue has a slow turnover, which records long-term dietary differences and reflects the entire feeding history of an animal [21]. Using this method, isotopic ratios are the result of a combination of the different food sources ingested during the entire life. Methods have recently been developed to delimit the range of possible proportion of various food items in an individual [22]. However, it is always necessary to have isotopic values for the range of all possible food items; a fact lacking in the current study because mole crickets appear to be opportunistic omnivores. Moreover, limited information is available about the fractionation processes responsible for the isotopic enrichment through metabolic pathways [23]. A difficulty for evaluating the incorporation of resources from mixed diets is that the isotopic signature may not exactly correspond to the signature of the food resource (see [24,25]). This lack of information impairs attempts to evaluate the absolute position of consumers in the food webs [26].

DeNiro and Epstein [27] tested experimentally the isotopic enrichment in the cuticle of the grasshopper *Melanoplus *sp.. They did not obtain the 3.4‰ expected increase of δ^15^N that is generally observed between two trophic levels, but rather observed a depletion. As a consequence, it seems that stable isotopic ratios in chitin are no longer used as a method to assess the position of an insect in a food web. The cuticle, however, is an inert tissue that reflects relative dietary variations during the growth period, and it retains this information in a chronological manner at each molt [28]. Hence our focus in this study was the analysis of the isotopic ratio variability within- and between age groups of the two study sites.

At the habitation site, the homogeneous carbon and nitrogen signals (δ^13 ^C and δ^15^N) obtained for the different mole cricket stages are interpreted as the isotopic signature of a mixed diet on a limited range of foods. The difference is striking when compared to the results obtained from mole crickets collected at the beach. The standard deviations in this sample suggest that the individual diets are heterogeneous.

The observed shift toward isotopic ratios of enriched marine resources is consistent with previous studies [28,29] which showed that stable-carbon isotope ratio can be enhanced by about 7‰. The positive correlation observed between δ^15 ^N ratios and the amount of organic matter in the beach confirms that mole crickets exploit this food resource during the nesting season. Inversely at the habitation site, the correlation test rejected the hypothesis of an arrival of mole crickets adults from the beach that would have fed on turtle eggs. Carbon and nitrogen stable isotopes of the cuticle appear in this context as markers of distinct feeding locations and it would allow to test the source-sink population hypothesis of a cyclic colonization in ephemeral habitats [30].

## Conclusion

From the wide range of isotopic signatures observed at the Awala-Yalimapo beach, it can be concluded that some mole crickets, but not all of them, feed on turtle eggs. The changes observed in the isotopic signatures resulted in the increasing mobility of the older mole crickets [31]. As *S. didactylus *increases in age it digs deeper into the soil and is more liable to encounter turtle eggs. Werner et al. [31] concluded that the consumption of a relatively limited fraction of the available resources was a means of feeding efficiency, *i.e*. nitrogen rich food for reproductive investment. Generalist insects are able to balance their food supply by ingestion of food materials complementing each other [32]. From these results it cannot be concluded that the individuals settled in the beach are specialist feeders that evolved adaptations to use marine resources. As such, they do not constitute a sub-population exploiting a particular ecosystem.

## Methods

### Collection of material

Fieldwork was conducted at the Amana Natural Reserve in French Guiana, during the 2002 nesting season of the marine leatherback turtle (*Dermochelys coriacea*). Nesting activity was monitored by counting tracks in the morning, or by female counts during the night. Data were available from March until July and during February.

Mole crickets (*Scapteriscus didactylus*) were caught at two locations: the Awala-Yalimapo beach (3.6 km long) and in the vicinity of Awala habitations. Inhabitants of Awala and Yalimapo villages are settled on the offshore cordon, a few hundred meters back from the beach. Given that dispersal flight for mating occurs in the winged species of *Scapteriscus *sp., mole crickets from the two locations are considered as belonging to the same population [33]. Juveniles, with limited mobility, explore their habitat by way of sub-surface galleries that are a few meters long. Often such galleries come upon marine remains at high-water marks. Remains on the sea strand were mostly drifting trees and plants from the Mana and Maroni rivers; very few dead marine animals were observed and no seaweeds.

As their development progresses, the mole crickets explore larger soil volumes digging deeper tunnels (see [1,11]). They live mostly upward of the high-water mark on the fore-dune, at the boundary of herbaceous vegetation, where they have at their disposal roots and leaves of dicotyledonous plants (convolvulaceae) and Gramineae.

Mole crickets were searched for along the beach at night, two hours after sunset, when they actively borrow subsurface tunnels in the sand. They were dug out of their galleries by hand. Three age classes, irrespective of the sex, were recorded: very young mole crickets (juveniles I) with alar rudiments in a downward position, late instars (juveniles II_R _+ III_R_) with pterotheca reversed upward and adults as a third class. Sixty-nine adults and 31 juveniles samples were taken from the beach before and during the turtle nesting season (from March to July) and during February. In the village, 41 adults and 25 juveniles of all ages were caught in wet sand near four kitchens garbage (from March to May then during November and February). All individuals were stored in 95% ethanol until analysis at the laboratory.

Potential food sources for the mole crickets were collected at the habitation sites and in the beach: food is traditionally prepared outside the house in a cooking area. Alimentary scraps and permanent humidity attract *S. didactylus *and *S. borelli*, and subsequently breed in such places. Traditional cooking is based on rice, cassava, fish and chicken year round. Turtles and turtle eggs are not cooked at Awala-Yalimapo villages. Poaching of turtle eggs is strictly forbidden in the Amana Natural Reserve and they are guards in charge of the turtle survey.

Potential food sources chosen at the habitation site were chicken meat and the muscles of a catfish (*Hexanematichthys couma*) cooked by the inhabitants and occasionally found dead on the sea strand. Samples were stored at -40°C and freeze-dried prior to the analysis. Some rice and cassava were also analyzed.

A sample of four leatherback turtle eggs were collected from the beach. Yolk and albumin were prepared separately, stored at -40°C and freeze-dried. Roots of *Ipomoae pes-caprae*, *Ipomoea stolonifera *and two undetermined gramineae were dried and ground prior to the analysis.

### Sample preparation and isotopic analysis

The foreleg tibias of the mole crickets were dissected and muscles were hydrolyzed overnight in a solution of potassium hydroxyde at 10%. Tibias were cleared of any muscle traces under a stereomicroscope, washed twice in de-ionized water and dried for 12 hours in an oven at 60°C. They were ground to a fine powder with a ball mill (Type MM200, Retsch, Haan, Germany). Turtle eggs and marine catfish were freeze-dried and ground. About 0.8 to 1.2 mg was analyzed for carbon and nitrogen determinations. Samples of 1 to 3 mg of plants were washed in de-ionized water, dried for 12 hours at 60°C in an oven, ground and analyzed for δ^13^C and δ^15^N. Organic matter was combusted in an elemental analyzer (Model NA-1500, Carlo Erba, Milan Italy) and the CO_2 _and N_2 _gases obtained were then analyzed using a stable isotope ratio mass spectrometer (VG Optima, Fisons, Villeurbanne, France) in order to determine ^15^N and ^13^C isotopic values [34]. Isotopic signatures are expressed in δ notation as ratios relative to PeeDee limestone (carbon) and the atmospheric N_2 _(nitrogen) standards as follows:

δX = 1000(R_sample _- R_standard_/R_standard_)

Where X is ^13^C or ^15^N and R is the corresponding ratio ^13^C/^12^C or ^15^N/^14^N.

The seven smallest and highest values obtained were duplicated twice for verification.

### Statistical analysis

ANOVA on δ^13^C and δ^15^N values were conducted with StatView Abacus Concept. Analysis of isotopic temporal variation was conducted using a Generalized Linear Model (GLM) with Julian day of capture (D) and location (L) (beach or habitation) as cofactors [see Additional file 1]. Day 0 was defined the 1^st ^of March of the year when the first leatherback turtles arrived to nest. Gaussian link function was used with mean value modeled as a polynomial function of order 0, 1 or 2 according to D. Standard Deviation has been modeled as *B*_1 _*Y*_*i *_+ *B*_0 _with *B*_*x *_fitted parameters and *Y*_*i *_the observed isotopic value for individual *i*. Maximum likelihood fit criteria has been used and model selection has been performed using Akaike Information Content [35]:

AIC = -2 ln *L *+ 2 *p *with *p *the number of parameters used for the model which is, by short, a measure that takes into account the quality of fit (-2 ln *L*) and penalizes a too high number of parameters used for the fit (2 *p*). The probability that a particular model is the best one among the set of tested model was calculated based on Akaike Weight [36].

### Leatherback nesting season

The nesting season of the leatherback turtle typically shows a peak of nesting at approximately the middle of the season. It rises to a maximum of about 200 nests per night in June. The number of nests before and after the nesting season is very low, generally less than one nest per week or even per month in some cases. Bouchard and Bjorndal [2] estimated the content of organic matter in eggs of loggerhead turtles as 0.126 g of dry organic matter per g of fresh egg. Using a cross-product with the weight of leatherback eggs (85 g) (see [37]) and taking into account the mean number of eggs in one nest (88 eggs), the average contribution to the beach of one leatherback nest is estimated to be 942.48 g of dry organic matter. The mass of organic matter from marine ecosystem deposited each month on the beach is then calculated multiplying the contribution of the nest with the total number of nests. The δ^13^C and δ^15^N signatures for mole crickets caught in the beach or at the habitations have been correlated using to the total weight of organic matter from marine origin deposited in the beach during the month of capture. Spearman non-parametric rank correlation has been used to take into account time is rendered discreet.

## Abbreviations

None

## Authors' contributions

AM collected the data, performed most of the biometric and biochemical analyses, participated in the design of the study and wrote the first draft as a Master thesis. CL was in charge of the isotopic analyses. MG and AL conceived of the study, participated in its design and coordination and wrote the final draft for submission to BMC. Fourth authors read and approved the final manuscript.

## Supplementary Material

Additional File 1 Model selection for δ 15N and δ 13C in cuticle of mole cricket caught on the beach and near habitations. Table of model selection for δ 15N and δ 13C in cuticle of mole cricket caught on the beach and near habitations. Click here for file

## Figures and Tables

**Table 1:** Carbon and nitrogen isotopic ratios δ^13^C and δ^15^N) of potential food resources for *Scapteriscus didactylus*. Plant and animal materials were sampled on the Awala-Yalimapo beach from March to July and February; at the habitation site, potential food resources of the mole cricket were chosen from food cooked by the inhabitants. SD = standard deviation, n = number of measures, nd = not detectable, na = not available

Food source		δ^15^N (‰)		δ^13^C (‰)	
	n	Mean	SD	Mean	SD
**Terrestrial food**					
Gramineae sp. roots	11	3.08	2.07	-15.58	5.07
Dicotyledons roots					
*Ipomoeae pes-caprae*	13	6.49	2.70	-25.70	1.64
*Ipomoeae stolonifera*	13	4.12	2.00	-26.91	1.31
Rice	3	2.27	0.04	-28.00	0.08
Cassava	5	nd	na	-25.74	0.38
Chicken meat	1	2.41	na	-20.39	na
**Marine food**					
Marine catfish	2	13.23	0.21	-14.55	0.12
Yolk of turtle eggs	4	10.53	0.14	-22.58	0.56
Albumin of turtle eggs	9	10.10	0.25	-16.57	0.42

**Table 2:** Comparison of the isotopic signatures δ^15^N and δ^13^C, measured in the cuticle of *Scapteriscus didactylus*. CV = 100*SD/Mean.

	n	Mean	Min	Max	SD	CV
**Habitations**						
**δ^15^N (‰)**						
Juveniles I	9	6.60	5.38	8.04	0.89	13.5
Juveniles IIR+IIIR	16	7.06	5.71	9.28	1.07	15.2
Adults	41	7.40	3.05	12.62	1.88	25.4
**δ^13^C (‰)**						
Juveniles I	9	-25.15	-25.32	-24.81	0.43	1.7
Juveniles IIR+IIIR	16	-25.15	-26.08	-24.25	0.51	2.0
Adults	41	-25.46	-27.46	-23.05	1.07	4.2
**Beach**						
**δ^15^N (‰)**						
Juveniles I	12	5.65	3.31	8.24	1.65	29.2
Juveniles IIR+IIIR	19	5.12	1.32	8.07	2.00	39.1
Adults	69	7.04	1.96	13.68	2.93	41.6
**δ^13^C (‰)**						
Juveniles I	12	-24.41	-25.45	-22.69	0.67	2.7
Juveniles IIR+IIIR	19	-24.74	-25.99	-22.62	1.27	5.1
Adults	69	-23.14	-26.31	-13.48	2.42	10.7
