# Supplementary material for: Evidence of the exploitation of marine resource by the terrestrial insect Scapteriscus didactylus through stable isotope analyzes of its cuticle
Source: BMC Ecol. 2006 May 8;6:6. doi: 10.1186/1472-6785-6-6 (PMC1533807; doi:10.1186/1472-6785-6-6)
Supplement: Additional File 1 — Model selection for δ 15N and δ 13C in cuticle of mole cricket caught on the beach and near habitations. Table of model selection for δ 15N and δ 13C in cuticle of mole cricket caught on the beach and near habitations. [file 1472-6785-6-6-S1.pdf]

Annex

Model selection for δ 15N and δ 13C in cuticle of mole cricket caught on the beach and near habitations. Polx designates a polynomial function of order x according to Julian date for the mean value of isotope ratio and H or C indicates whether the standard deviation is modeled as a constant (C) or dependent on the observed value for isotope ratio (H for heteroskedasticity). Selected model based on AIC is shown in bold. The best specific model AIC is the sum of the AIC for selected model for beach and habitation.

**δ ^{15}N**	**Beach**			**Habitations**			**Common model**		
**Model**	**-Ln L**	**AIC**	**Akaike weight**	**-Ln L**	**AIC**	**Akaike weight**	**-Ln L**	**AIC**	**Akaike weight**
**Pol2 H**	192.19	**394.39**	**0.972**	80.64	171.28	0.089	281.06	572.13	0.010
**Pol2 C**	203.09	414.18	0.000	80.93	169.86	0.182	297.4	602.81	0.000
**Pol1 H**	196.75	401.5	0.028	82.1	172.2	0.056	287.43	582.86	0.000
**Pol1 C**	203.43	412.88	0.000	81.95	169.91	0.177	287.43	580.86	0.000
**Pol0 H**	203.79	413.58	0.000	82.24	170.48	0.133	298.63	603.26	0.000
**Pol0 C**	203.79	411.58	0.000	82.24	**168.48**	**0.362**	298.63	601.26	0.000
							**Best specific model**	**562.87**	**0.990**

$\delta^{13}C$	Beach			Habitations			Common model		
**Model**	**-Ln L**	**AIC**	**Akaike weight**	**-Ln L**	**AIC**	**Akaike weight**	**-Ln L**	**AIC**	**Akaike weight**
**Pol2 H**	156.77	**323.54**	**0.9272**	58.24	126.48	0.099	241.15	492.31	0.000
**Pol2 C**	164.36	336.72	0.0013	58.48	124.96	0.213	248.7	505.4	0.000
**Pol1 H**	160.4	328.8	0.0668	59.85	127.7	0.054	240.86	489.73	0.000
**Pol1 C**	164.68	335.36	0.0025	59.9	125.8	0.140	248.87	503.74	0.000
**Pol0 H**	166.15	338.3	0.0006	59.95	125.9	0.133	253.81	513.63	0.000
**Pol0 C**	166.15	336.3	0.0016	59.95	**123.9**	**0.361**	253.81	511.63	0.000
							**Best specific model**	**447.44**	**1.000**
